# A 3-mm protrusion threshold after pediatric forearm ESIN: a rule-out safety margin, not a decision rule

**DOI:** 10.2340/17453674.2026.46139

**Published:** 2026-06-12

**Authors:** Rui ZHANG, Sheng LU

**Affiliations:** 1 Department of Orthopedic Trauma, Qujing Central Hospital of Yunnan Province/Affiliated Qujing Hospital of Kunming Medical University, Yunnan Engineering Research Center for Precision Medical Testing, Qujing, Yunnan Province;; 2 Department of Orthopedics, the First People’s Hospital of Yunnan Province, the Affiliated Hospital of Kunming University of Science and Technology, Kunming, Yunnan Province;; 3 The Key Laboratory of Digital Orthopedics of Yunnan Province, Kunming, Yunnan Province;; 4 Intelligent Orthopedic Medical Technology Research Center, Kunming University of Science and Technology, Kunming, Yunnan Province, China

*Sir,—*We read with great interest the study by Brattgjerd et al. on the long-term retention of implants after impacted ESIN placement for pediatric forearm diaphyseal fractures [1]. They explored whether implant retention could be a safe alternative to routine nail removal when nail protrusion is kept to a minimum. ESIN is now widely used in the treatment of pediatric forearm fractures [2] and implant removal may be associated with complications [3]. Their data confirms that impacted ESIN is good treatment, but the 3.3 mm threshold appears more useful for excluding irritation-related implant removal than for predicting it.

The authors’ data shows that when nail protrusion was less than 3.3 mm, there were no cases of implant removal due to irritation (negative predictive value: 100%) [1]. A post hoc “rule-of-three” calculation indicates that the upper limit of the 95% confidence interval for the irritation-related removal rate is about 2.3% (3/133 nails). When protrusion is higher than this threshold, the positive predictive value is only 16% [1]. This means that most nails with protrusion exceeding 3.3 mm can still be retained, indicating that the threshold functions to define a low-risk zone rather than identify symptomatic nails.

In clinical practice, the ROC cut-off value can maximize the Youden index, but cannot define the optimal clinical management plan [4]. If the 3.3 mm cut-off is translated too rigidly into an intraoperative target of around 3 mm, it may lead to efforts to minimize nail protrusion during surgery; however, when too deeply positioned nails need to be removed later because of refracture, infection, local discomfort, or family preference, later extraction may become more difficult. Brattgjerd et al. reported a case of failed removal of a firmly positioned ulnar nail with distal perforation [1]. Existing evidence shows that implant retention time and protrusion degree may affect the difficulty of removal [5]. Clinical decision-making needs to strike a balance between avoiding soft-tissue irritation and ensuring the safety of subsequent removal operations.

Local irritation symptoms are not determined only by the extraosseous length of the nail. In the study, 3.8% of patients developed delayed discomfort, most of whom had no measurable nail protrusion [1]. This suggests that factors such as soft-tissue coverage, nail entry site, bone involved, patient activity level, and growth-related changes may all modify the relationship between radiographic protrusion and symptoms.

The clinically useful message may therefore be: if implant retention is intended, nail prominence should be minimized intraoperatively, preferably to below approximately 3 mm when safely achievable; however, postoperative implant removal should remain symptom-driven rather than threshold-driven.


**Rui ZHANG ^1^ and Sheng LU ^2–4^** *^1^ Department of Orthopedic Trauma, Qujing Central Hospital of Yunnan Province/Affiliated Qujing Hospital of Kunming Medical University, Yunnan Engineering Research Center for Precision Medical Testing, Qujing, Yunnan Province;* *^2^ Department of Orthopedics, the First People’s Hospital of Yunnan Province, the Affiliated Hospital of Kunming University of Science and Technology, Kunming, Yunnan Province;* *^3^ The Key Laboratory of Digital Orthopedics of Yunnan Province, Kunming, Yunnan Province;* *^4^ Intelligent Orthopedic Medical Technology Research Center, Kunming University of Science and Technology, Kunming, Yunnan Province, China* *Correspondence: Sheng Lu, drlusheng@163.com*

